# Antifungal and Antibiofilm Activities and the Mechanism of Action of Repeating Lysine-Tryptophan Peptides against *Candida albicans*

**DOI:** 10.3390/microorganisms8050758

**Published:** 2020-05-18

**Authors:** Gopal Ramamourthy, Jonggwan Park, Changho Seo, Hans J. Vogel, Yoonkyung Park

**Affiliations:** 1 Biochemistry Research Group, Department of Biological Sciences, University of Calgary, Calgary, AB T2N 1N4, Canada; rgopal@ucalgary.ca (G.R.); vogel@ucalgary.ca (H.J.V.); 2 Department of Biomedical Science and BK21-Plus Research Team for Bioactive Control Technology, Chosun University, Gwangju 61452, Korea; 3 Department of Bioinformatics, Kongju National University, Kongju 38065, Korea; for_quality@naver.com (J.P.); chseo@kongju.ac.kr (C.S.); 4 Research Center for Proteineous Materials, Chosun University, Gwangju 61452, Korea

**Keywords:** lysine, tryptophan, antifungal, antibiofilm, RNA binding, fungicidal activity

## Abstract

The rapid increase in the emergence of antifungal-resistant *Candida albicans* strains is becoming a serious health concern. Because antimicrobial peptides (AMPs) may provide a potential alternative to conventional antifungal agents, we have synthesized a series of peptides with a varying number of lysine and tryptophan repeats (KW_n_-NH_2_). The antifungal activity of these peptides increased with peptide length, but only the longest KW_5_ peptide displayed cytotoxicity towards a human keratinocyte cell line. The KW_4_ and KW_5_ peptides exhibited strong antifungal activity against *C. albicans*, even under conditions of high-salt and acidic pH, or the addition of fungal cell wall components. Moreover, KW_4_ inhibited biofilm formation by a fluconazole-resistant *C. albicans* strain. Circular dichroism and fluorescence spectroscopy indicated that fungal liposomes could interact with the longer peptides but that they did not release the fluorescent dye calcein. Subsequently, fluorescence assays with different dyes revealed that KW_4_ did not disrupt the membrane integrity of intact fungal cells. Scanning electron microscopy showed no changes in fungal morphology, while laser-scanning confocal microscopy indicated that KW_4_ can localize into the cytosol of *C. albicans*. Gel retardation assays revealed that KW_4_ can bind to fungal RNA as a potential intracellular target. Taken together, our data indicate that KW_4_ can inhibit cellular functions by binding to RNA and DNA after it has been translocated into the cell, resulting in the eradication of *C. albicans*.

## 1. Introduction

The increased incidence of infections caused by pathogenic yeasts that are resistant to commonly used antifungal drugs such as fluconazole has led to an urgent need for the development of new antifungal agents [1,2,3,4]. Moreover, fungal biofilms are also becoming recognized as a complicating factor that plays a major role in the clinical treatment of fungal infections [4,5,6]. The opportunistic pathogenic yeast *Candida albicans* is known to be the cause of most chronic infections in humans; for example, it plays a role in urinary tract infections, candidiasis and mycosis [2]. This organism can actively form biofilms, and hence the development of novel anticandidal agents that possess antibiofilm activity is a topic of intense research interest. 

Antimicrobial peptides (AMPs) are currently being considered as a promising alternative for antifungal drugs [6,7]. The majority of the antimicrobial peptides are highly cationic molecules, containing several lysine (Lys) and/or arginine (Arg) residues, and up to 50% hydrophobic amino acids. AMPs are generally distinguished by variations in their length, amino acid sequence, and structural properties (helical, beta-sheet, turns, extended, etc.) [8,9,10]. Most AMPs share amphipathic properties, which allows the cationic peptides to bind to negatively charged microbial membrane surfaces. Interestingly, beyond killing bacteria, many AMPs can also inhibit and eradicate pathogenic parasites, fungi and viruses [9,11]. Moreover, recent studies have indicated that many AMPs act as host-defense peptides that can stimulate the innate and adaptive immune responses of the host [12,13]. Some peptides have already been studied in clinical trials for the development of topical drugs against skin infections [14]. However, naturally occurring and synthetically obtained AMPs have so far proven challenging to turn into therapeutic compounds due to concerns about cytotoxicity, low stability, salt sensitivity and a high cost of production. Indeed, several AMPs have displayed significant toxicity for human cell membranes (e.g., pardaxin, melittin and several cathelicidins are cytotoxic due to their membrane lytic mechanism of action [15,16,17]). In addition, it is well known that physiological salt concentrations can sometimes reduce the effectiveness of cationic AMPs. Therefore, the synthesis of short AMPs that contain an appropriate mix of hydrophobic and cationic amino acids, and that have a low cytotoxicity and a reduced sensitivity to salt, is an important strategy that is being deployed in many laboratories to design clinically useful AMPs [18,19,20]. 

As mentioned above, most short AMPs contain several positively charged basic Arg and/or Lys residues that are thought to selectively mediate electrostatic interactions with negatively charged microbial membranes [21,22]. The guanidinium group of Arg can interact strongly with negatively charged phospholipid membranes, thereby promoting prokaryotic cell toxicity. However, strong interactions with the phosphodiester group in phospholipid headgroups can lead to cytotoxicity for Arg-containing AMPs. At the same time, the action of many AMPs is known to be dependent on Lys rather than Arg residues [23,24,25], and such Lys-containing AMPs are often less membranolytic. Studies of the cellular internalization of Lys-rich proteins have indicated that this basic amino acid can play an essential role in this process [26]. In some studies it has been shown that Lys containing synthetic peptides and RNA can bind to each other, a property that could be essential for their action in microbial cells and give rise to the inhibition of cellular protein synthesis, thereby leading to cell eradication [27]. The aromatic hydrophobic amino acids tryptophan (Trp) and phenylalanine are also frequently found in AMPs. The indole side chain of Trp can effectively interact with a negatively charged microbial membrane interface when compared with other nonpolar side chains (such as phenylalanine, which penetrates deeper into the membrane bilayer) [28,29]. As well, many biologically active peptides have been improved by introduction of a Trp residue in lieu of other hydrophobic amino acids [30,31,32]. Moreover, studies with short combinatorial cationic peptide libraries have highlighted a unique role for Trp residues in generating antibacterial activities [33]. This Trp-rich motif is also found in many naturally occurring peptides, such as AMPs that are derived from host defense proteins, such as lactoferrin or lysozyme [34]. 

**Figure 1:**
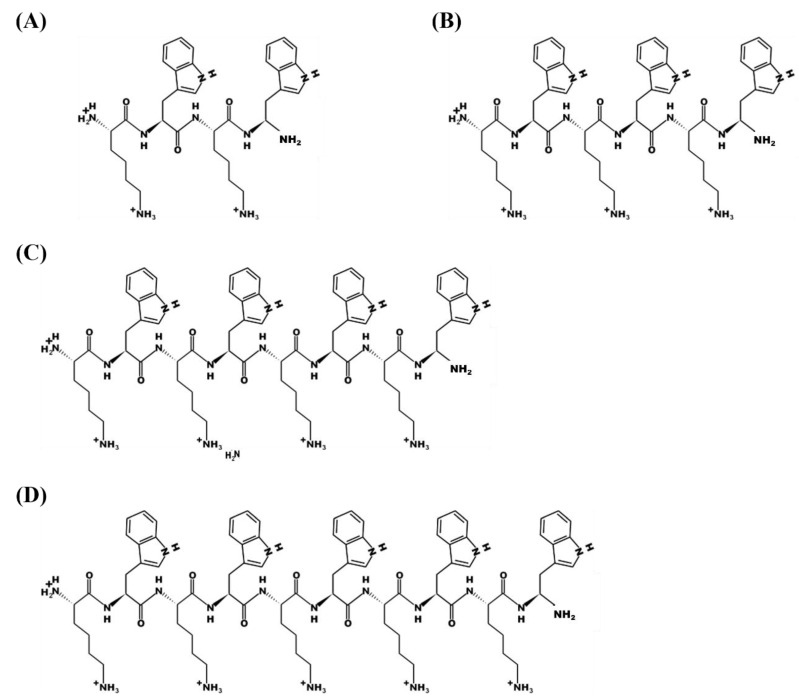
Structures of the peptides used in this study. (**A**) KW_2_, (**B**) KW_3_, (**C**) KW_4_ and (**D**) KW_5._

With all of these considerations in mind, we have designed the KW_n_ peptide series with repeated sequences and a balanced number of Lys and Trp residues that differ only in length of the peptide (Figure 1) [35]. In a previous study we established that octapeptides with the amino acid sequence KWKWKWKW-NH_2_ had significant antimicrobial activity that acted via a membrane disruption mechanism to break down the permeability barriers of both Gram-negative and Gram-positive bacteria [35]. In the present study, for the same group of peptides, we have found that the number of KW repetitions greatly impacts their antifungal activity, cytotoxicity and resistance to physiological salt concentrations. Moreover, by studying their membrane- and RNA-binding properties, as well as their membrane perturbation properties, we were able to determine a mechanism of action that contributes to fungal cell eradication.

## 2. Materials and Methods

### 2.1. Materials

For peptide synthesis, fluoren-9-ylmethoxycarbonyl (FMOC) amino acids, rink amide 4-methylbenzhydrylamine resin and other chemicals were purchased from Calbiochem-Novabiochem (La Jolla, CA, USA). Acrylamide, ergosterol and MTT were purchased from Sigma-Aldrich (St. Louis, MO, USA). Cholesterol (CH, from porcine liver), L-α-phosphatidylethanolamine (PE, from *Escherichia coli*), egg yolk L-α-phosphatidylcholine (PC), L-α-phosphatidylglycerol (PG, from *E. coli*), sphingomyelin (SM) and phosphatidylinositol (PI) were obtained from Avanti Polar Lipids (Alabaster, AL, USA). Calcein, 5-carboxytetramethylrhodamine (TAMRA), 3,3′-diethylthiodicarbocyanineiodide (DiSC_3_-5) and SYTOX Green and Propidium iodide (PI) were acquired from Molecular Probes (Eugene, OR, USA). All other reagents purchased were analytical grade. Double distilled water was used in the preparation of buffers (Millipore Co., Bedford, MA, USA). The fungal strains *Candida albicans* (*C. albicans*), *Candida catenulate* (*C. catenulate*), *Candida intermedia* (*C. intermidia*), *Candida rugosa* (*C. rugosa*), *Candida glabrata* (*C. glabrata*) *and Candida melibiosica* (*C. melibiosica*) isolates were obtained from the Korean Collection for Type Cultures (KCTC). Drug resistant *C. albicans* (CCARM 14001, CCARM 14007 and CCARM 140020) were collected from the Culture Collection of Antibiotic-Resistant Microbes (CCARM). 

### 2.2. Peptide Synthesis

The peptides KWKW-NH_2_ (KW_2_), KWKWKW-NH_2_ (KW_3_), KWKWKWKW-NH_2_ (KW_4_) and KWKWKWKWKW-NH_2_ (KW_5_) were synthesized by standard solid-phase methods using FMOC chemistry on a solid support of rink amide 4-methylbenzhydrydrylamine resin [35]. The crude peptides were resuspended in diethyl ether then purified by reverse-phase preparative HPLC on a Vydac C_18_ column (4.6 × 250 mm, 300 Å, 5 nm). The purified peptides displayed a sharp single peak; they were eluted within ~60 min using a 5–60% acetonitrile gradient in water containing 0.05% trifluoroacetic acid (*v*/*v*). The molecular masses of the purified peptides were confirmed by matrix-assisted laser desorption ionization mass spectrometry (MALDI II; Kratos Analytical Instruments). Peptide labeling at the N-terminal amino acid with 5-carboxytetramethylrhodamine (TAMRA) was done on the resin-bound peptide as previously described [36]. A KW6 peptide was also synthesized, but was poorly soluble in our buffer solutions, and hence not further analyzed.

### 2.3. Antifungal Activity

The fungal strains *C. albicans* (KCTC 7270), *C. catenulate* (7642), *C. intermidia* (7234), *C. rugosa* (7324), *C. glabrata* (7219) and *C. melibiosica* (KCTC 7631) were cultured at 28 °C in appropriate media. Fungal cells (final concentration 2 × 10^4^ cells/ mL) in 50 μL of YPD media (dextrose 2%, peptone 1% and yeast extract 0.5%) were seeded in each well of a microtiter plate containing 50 μL of two-fold serially diluted peptides in phosphate buffered saline. After incubation for 24 to 30 h at 28 °C, the lowest concentration of the peptides inhibiting the growth of the fungi was microscopically determined to be the minimum inhibitory concentrations (MICs) [37]. The effect of salt concentration on the antifungal activity of the peptides was tested by determining the MICs in the presence of NaCl and MgCl_2_. Fixed concentrations of NaCl (50, 100 and 150 mM) or MgCl_2_ (1, 2 and 5 mM) were added to the sodium phosphate buffer (pH 7.2). The effect of pH on the antifungal activity of the peptides was tested by determining peptide MICs in the presence of sodium phosphate buffer at a variety of pH values. 

### 2.4. Aggregation of KW_5_ Monitored by Thioflavin T (ThT) Fluorescence Assay

The effect of different buffers or pH values on the solubility of KW_5_ was determined using the ThT fluorescence assay. Different concentrations of KW_5_ were prepared with various buffer conditions (e.g., sodium phosphate buffer, pH 7.2; phosphate buffered saline, sodium phosphate buffer, pH 7.2 with 150 mM Nacl or 5 mm MgCl_2_; and sodium phosphate buffer, pH 5.2). Solutions of 20 μL of KW_5_ were mixed with 80 μL freshly prepared 5 μM thioflavin T (ThT) in an appropriate buffer solution. ThT fluorescence intensity was measured on a microplate spectrofluorometer (SpectraMax GeminiXS; Molecular Devices, Sunnyvale, CA, USA) using an emission wavelength of 490 nm with excitation at 445 nm.

### 2.5. Fungicidal Action

The activities of all the peptides against *C. albicans* were tested by using the standard microdilution plate candidacidal assay [38]. Mid-logarithmic growth phase *C. albicans* was grown in YPD medium then washed with PBS. The *C. albicans* cells (2 × 10^4^ cells/mL) were mixed at the MIC concentration of the peptides and incubated at 28 °C, aliquots were removed at fixed time internals, appropriately diluted and plated on YPD agar plates, then CFUs were counted after 16–24 h incubation at 28 °C.

To determine whether the candidacidal activity of these peptides is dependent on the metabolic activity of Candida, a fungicidal assay was performed as described above with peptides in the presence of 5 mM sodium azide, which blocks mitochondrial respiration [39]. In this experiment, cells were incubated with sodium azide (5 mM) for 30 min at 28 °C prior to the addition of the peptides. The optimal concentration of sodium azide (5 mM) was determined in preliminary experiments to avoid serious toxic effects of this compound on *C. albicans.*

### 2.6. Liposome Fusion Assay

Aggregation of lipid vesicles was monitored by absorbance measurements in PBS buffer at pH 7.2. Peptides (2.5, 5, 10, 20 and 40 μM) in PBS solutions were added to a suspension of 400 μM large unilamellar vesicles (LUVs) consisting of PC–CH–SM (1:1:1, *w*/*w*/*w*). An increase of absorbance indicates aggregation of the liposomes. Absorbance was measured at 405 nm using a microplate Autoreader EL 800 (Bio-Tek Instruments, Inc., Winooski, VT, USA) before and after the addition of peptide [40].

### 2.7. Calcein LEAKAGE

Calcein-entrapped LUVs composed of PE–PC–PI–ergosterol (5:4:1:2, *w*/*w*/*w*/*w*) or PC–CH–SM (1:1:1, *w*/*w*/*w*) were prepared by mixing the dried lipid in buffer solution (containing 70 mM calcein, PBS, pH 7.4). The suspension was freeze-thawed in liquid nitrogen for nine cycles and then extruded through two stacked 0.2-μM pore-size polycarbonate filters for 16 times using an Avanti Mini-Extruder (Avanti Polar Lipids Inc., Alabaster, AL, USA). Vesicles containing entrapped calcein were separated from free calcein by gel filtration chromatography on a Sephadex G-50 column. Entrapped LUVs in a suspension containing 2.5 μM lipids were then incubated with various concentrations of the peptide (0.0–0.25 μΜ) for 25 min. The release of calcein fluorescence was followed using a spectrofluorometer (Perkin-Elmer LS55, Mid Glamorgan, UK) with appropriate wavelengths (excitation, 480 nm; and emission, 520 nm). The calcein was completely (100%) released after the addition of 0.1% Triton X-100. Spontaneous leakage was determined to be negligible on this timescale. The experiments were conducted at 25 °C. The percentage of calcein release was calculated according to the following equation [41]:

Release (%) = 100 × (*F − F_o_*)/(*F_t_ − F_o_*) (Ex. 480 nm and Em. 520 nm) (1)

where *F* represent the fluorescence intensity before the addition of the detergent, *F_t_* represents the fluorescence intensity after the addition of the detergent and *F_o_* represents the fluorescence of the intact vesicles.

### 2.8. Circular Dichroism (CD) Spectroscopy

Circular dichroism (CD) spectra were recorded between 190 and 250 nm on a Jasco 810 spectropolarimeter (Jasco, Tokyo, Japan) equipped with a temperature control unit, using a 0.1-cm path-length quartz cell at 25 °C. The CD spectra were measured for peptide samples (50 μM) that were dissolved in PBS buffer (pH 7.2) and PBS containing 1 mM PE–PC–PI–ergosterol (5:4:1:2, *w*/*w*/*w*/*w*) or PBS containing 1 mM PC–CH–SM (1:1:1, *w*/*w*/*w*) vesicles. The CD spectrum for each peptide was obtained by averaging the values from three independent recordings with four scans each.

### 2.9. Cytotoxicity

We examined the cytotoxic effects of the peptides using HaCaT (human keratinocyte) cells, which were cultured in Dulbecco’s modified Eagle medium (DMEM) supplemented with antibiotics (100 U/mL penicillin, 100 µg/mL streptomycin) and 10% fetal calf serum at 37 °C in a humidified chamber with atmosphere containing 5% CO_2_. To measure viable cells and growth inhibition, an MTT colorimetric assay was used. A total of 4 × 10^3^ cells was seeded per well into a 96-well plate incubated at 37 °C for 24 h. The next day, cells from plates were treated with various concentrations of the tested peptides. After 24 h incubation at 37 °C, MTT (10 μL) was added at a concentration of 5 mg/mL to each well. The culture supernatants were discarded after a further 4 h, and the remaining precipitate was dissolved by the addition of 100 μL of DMSO into the wells. Absorbance was then measured at a wavelength of 570 nm using an EL800 reader (Bio-Tek instruments, Inc., Winooski, VT, USA).

### 2.10. Biofilm Inhibition Assays

To test biofilm inhibition by the peptides, 180 μL (1 × 10^6^ cells/mL) of *C. albicans* suspensions in RPMI supplemented with 0.2% glucose and 20 μL of peptide at concentrations ranging from 12.5 to 100 μM were added into the plate, and the mixtures were incubated for 24 h at 28 °C. The supernatant was removed, and the biofilms were fixed by adding 100% methanol. After 10 min, the methanol was removed, and the samples were dried at room temperature. The biofilms formed in the wells were stained with 0.1% (*w*/*v*) crystal violet (Sigma-Aldrich, Incheon, Korea) for 20 min and then washed with distilled water until the control had a transparent color. Lastly, stained biofilms were dissolved in 95% ethanol to measure absorbance at 595 nm using a Versa-Max microplate ELISA reader (Molecular Devices, Sunnyvale, CA, USA). The percentage biofilm mass was calculated using the equation (A_595_ of the treated biofilm/A_595_ of the untreated biofilm) × 100 [42,43]. The images were observed by fluorescent microscopy (Olympus IX71; Tokyo, Japan) using an EVOS FL Auto 2 imaging system (Invitrogen, Waltham, MA, USA).

### 2.11. Cell Wall Binding

The effect of fungal cell wall components on the anticandidal activity of KW_4_ was examined using the microtiter plate candidacidal assay as well as the ultrasensitive radial diffusion assay [38,44]. Peptide at the MIC concentration was incubated with different concentrations of cell wall components (laminarin or mannan (0.5–8 mg/mL)) for 1 h at 37 °C. The effect of cell wall components on the fungicidal effect of KW_4_ was assessed by the microdilution plate candidacidal assay as described above. 

For the radial diffusion assay, 20 µL of a KW_4_ solution (final concentration = 8 µM) was added to 80 µL of each polysaccharide (0.5–8 mg/mL in PBS, pH 7.2). including laminarin (Beta-1,3-glucan polymer; Sigma-Aldrich, Incheon, Korea) or mannan (mannose polymer; Sigma-Aldrich, Incheon, Korea), and incubated with *C. albicans* for 1 h at 37 °C. Eight microliter samples were impregnated into wells (3 mm diameter) that had been punched in underlay gels containing about 2 × 10^4^ cells/mL of *C. albicans*. The underlay agars consisted of PBS, 1% *w*/*v* agarose (Sigma, Incheon, Korea), and 0.3 mg of YPD/mL. After 3 h of incubation at 37 °C, a 10-mL overlay gel of 1% agarose and 6% YPD was poured on the underlay agarose gel. The plates were incubated overnight at 37 °C to allow surviving *C. albicans* to form detectable colonies on solidified agars, but no colonies in the inhibition zone indicated antifungal activity. Experiments were performed in triplicate. Next, the CD spectra of the peptides were monitored at a concentration of 50 μM of KW_4_ in buffer in the presence of laminarin (0.05%) or mannan (0.05%).

### 2.12. SYTOX Green

Membrane permeabilization of *C. albicans* was studied using the fluorescent dye SYTOX Green (Molecular Probes, Eugene, OR, USA). Fungal cells were grown to mid-logarithmic growth phase then centrifuged, washed and resuspended in PBS. Cells were suspended in PBS (2 × 10^7^ cells/mL) and incubated with 1 µM SYTOX Green for 15 min in the dark prior to the influx assay. At 2–3 min after initiating data collection, 1/2× or 1× MIC peptides were added to the cell suspensions. Due to the binding of the dye to intracellular DNA, the increases in SYTOX Green fluorescence was measured for 120 min (excitation and emission wavelengths were 485 nm and 520 nm, respectively). Lysis of the cells in Triton X-100 (0.1%) or melittin gave maximum fluorescence.

### 2.13. Flow Cytometry

*C. albicans* (KCTC 7270) was cultured in YPD medium then harvested by centrifugation (4000 rpm for 10 min). The harvested *C. albicans* cells were washed three times with PBS and resuspended (2 × 10^4^ cells/mL) in PBS. Afterwards, PI (10 μg/mL) was added to the *C. albicans* suspensions, then incubated with peptides or with PBS used as the control for 10, 20 and 30 min at 28 °C. The suspensions were centrifuged at 4000 rpm for 10 min and subsequently washed with PBS to remove any unbound PI. The data were analyzed by using a CytoFLEX flow cytometer (Beckman Coulter, Brera, CA, USA). 

### 2.14. Fluorescence Microscopy Analysis of Cell Permeabilization

The cell permeability was monitored using the DNA-staining fluorescent probe PI as previously described [45]. For this experiment, *C. albicans* cells were grown in YPD broth with shaking at 200 rpm overnight at 28 °C. Cells were harvested by centrifugation (4000 rpm for 10 min), washed and suspended in PBS to yield 2 × 10^6^ cells/mL; 100 μL of *C. albicans* suspensions (2 × 10^6^ cells/mL) were incubated with 5 μL from respective peptide aqueous stock solutions to yield a final peptide concentration of 8 μM KW_4_ peptide or 4 μM melittin (as a control peptide) for 10, 20 and 30 min at 28 °C. Next, PI was added to each sample at a final concentration of 1 μg/mL and the plate was incubated at 28 °C for 5 min. The effects of the peptides on *C. albicans* were visualized by the EVOS FL Auto 2 imaging system (Invitrogen, Waltham, MA, USA). Cells without added peptide served as a control.

### 2.15. Scanning Electron Microscopy

A mid-logarithmic phase culture of *C. albicans* cells was re-suspended at 2 × 10^7^ cells/mL in PBS (pH 7.2) and incubated at 37 °C with a final concentration of 12.5 μM of KW_4_. After 60 min, the cells were pelleted by centrifugation at 3000× *g* for 5 min, then washed twice in PBS. The supernatants were removed, and the pellets were fixed in 500 μL of 5% (*v*/*v*) glutaraldehyde in 0.2 M sodium-cacodylate buffer (pH 7.4). After fixation for 3 h at 4 °C, the samples were extensively washed with 0.1 M sodium-cacodylate buffer. The samples were treated with 1% (*w*/*v*) osmium tetroxide in 0.1 M sodium-cacodylate buffer in the dark for 1 h at 4 °C. The cells were then washed twice in 5% (*w*/*v*) sucrose in the same buffer and dehydrated in 20%, 40%, 60%, 80%, 95% and 100% ethanol, sequentially. After lyophilization and coating, the samples were examined using a scanning electron microscope (Hitachi S-2400N, Tokyo, Japan). A control image was obtained in the absence of peptide solution.

### 2.16. Tryptophan Fluorescence Spectroscopy and Acrylamide Quenching Assay

The Trp fluorescence emission spectra of the peptides were monitored in 10 mM sodium phosphate buffer in the presence of small unilamellar vesicles (SUVs) composed of either PE–PC–PI–ergosterol (5:4:1:2, *w*/*w*/*w*/*w*) or PC–CH–SM (1:1:1, *w*/*w*/*w*). In these studies, SUVs were used in order to avoid or minimize differential light scattering effects [46]. The Trp fluorescence measurements were made using a spectrofluorometer (Perkin-Elmer LS55, Mid Glamorgan, UK). Each peptide was added to 1 mL of PBS (pH 7.2) containing 200-μM liposomes, and the peptide–liposome mixture (a molar ratio of 1:100) was allowed to interact for 10 min at 25 °C. The Trp fluorescence was excited at 280 nm, and the emission was scanned from 300 to 400 nm.

The fluorescence quenching experiments were performed using acrylamide as the Trp fluorescence quencher, the concentration of which was between 0.04 to 0.20 M in the cuvette. The effect of acrylamide on the fluorescence of each peptide was recorded and then analyzed with the Stern–Volmer equation:

*F*_0_/*F* = 1 + *K*_SV_ (Q) (2)

where *F*_0_ is the fluorescence intensity in the absence of acrylamide, *F* represents the fluorescence intensity in the presence of acrylamide, *K*_SV_ is the Stern–Volmer quenching constant and (Q) is the concentration of the Trp fluorescence quencher.

### 2.17. Nucleic Acid Binding

Briefly, RNA was extracted from *C. albicans* using the TRI reagent (Molecular Research Center, Cincinnati, OH, USA) according to the manufacturer’s instructions. Purified RNA was collected and resuspended in RNAse-free water. Then, the yeast RNA (10 µg) was mixed with peptide at different concentrations, 2.5–30 µg in 15 µL of 10 mM Tris, 1 mM EDTA buffer (pH 8.0). After 10 min incubation at room temperature, the solution was subjected to electrophoresis on a 1% agarose gel in Tris Borate EDTA (TBE) buffer. Gel retardation was visualized under UV illumination using a Bio-Rad Gel Documentation system.

### 2.18. Confocal Laser-Scanning Microscopy (CLSM)

*C. albicans* cells were collected from mid-logarithmic phase and washed with PBS (pH 7.2). After incubation with rhodamine-KW_4_ (8 µM/mL) at 28 °C for 10 min, the cells were washed with buffer then immobilized on a glass slide. The accumulation of the rhodamine-labeled peptides in the cells was observed with an Olympus FV1000 confocal laser-scanning microscope (Tokyo, Japan).

## 3. Results

### 3.1. Effect of the Chain Length of the KW_n_ Peptides on Their Antifungal Activity

Table 1 shows the MIC values determined for the KW_n_ peptide series against *C. albicans,* including several fluconazole-resistant *C. albicans* strains. The MIC values clearly decreased with increases in the chain length of the peptides. The shortest KW_2_ peptide was inactive, but the KW_3_, KW_4_, and KW_5_ peptides all possessed antifungal activity, with MICs in the micromolar range. Interestingly, the KW_4_ and KW_5_ peptides had a similar antifungal activity against the regular *C. albicans* strain. However, for the fluconazole-resistant strains, KW_3_ appears to have been inactive, while both the KW_4_ and KW_5_ peptides maintained most of their activity (as evidenced by their MIC values). A substantial increase in the activity of KW_5_ against fluconazole-resistant *C. albicans* strains when compared to the KW_4_ peptide was noted, as shown in Table 1. We also found that the KW_3_, KW_4_ and KW_5_ peptides had antifungal activity against other *Candida* species such as *Candida catenulate, Candida rugosa*, *Candida melibiosica, Candida glabrata* and *Candida intermedia* (see Supplementary Table S1).

### 3.2. Examining the Influence of Salt and pH on the KW_5_ Activity and Self-Association in Aqueous Solution

**Figure 2:**
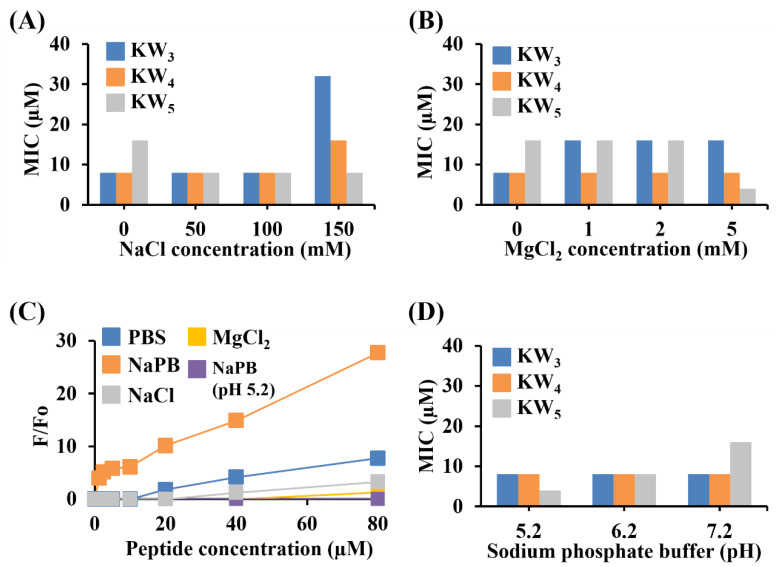
Effect of peptide length, salt and pH on the MICs of the KW_n_ peptides against *C. albicans* and self-association of the peptide in aqueous solutions. MICs of the peptides against *C. albicans* in the presence of 10 mm NaPB supplemented with (**A**) NaCl and (**B**) MgCI_2_. (**C**) ThT (20 μM) fluorescence emission intensity measured for KW_5_ in different buffer conditions. (**D**) Effect of pH on the antifungal activity of the KW_n_ peptides. An average of all values was obtained from three experiments in duplicate. The data of all test samples were obtained from three replicates, and error bars represent the SD. Data points without error bars indicate that the SD is small and not visible.

As expected from the results obtained earlier in antibacterial assays, our results show that the activities of KW_3_, KW_4_ and KW_5_ against *C. albicans* could be reduced somewhat by increasing salt concentrations (NaCl and MgCl_2_). While the antifungal activity of KW_3_ against *C. albicans* was dramatically decreased by increasing salt concentrations from 0 to 150 mM NaCl or from 0 to 5 mM MgCl_2_, KW_4_ did not experience a reduced activity in 100 mM NaCl or 5 mM MgCl_2_. However, its activity decreased two-fold in 150 mM NaCl, when compared to 100 mM NaCl. Increasing the salt concentrations or decreasing the buffer pH also influenced the antifungal activity of KW_5_, as evidenced from a two-fold increase in activity of KW_5_ in higher salt conditions (150 mM NaCl and 5 mM MgCl_2_) or a lower pH 5.2 (Figure 2A–C). In contrast, however, the antifungal activities of KW_3_ and KW_4_ were not altered by changes in the pH. These observations suggest that KW_5_ is likely aggregated in low salt concentrations or at higher pH values (pH 7.2 or 6.2), but that this aggregated state can be dissociated by higher salt conditions or at a lower pH 5.2 (Figure 2C). The self-aggregation status of KW_5_ was therefore examined under different conditions by using thioflavin-T (ThT), a dye that is known to increase in fluorescence upon binding to β-sheet peptides or amyloids (Figure 2C). The results show that the ThT fluorescence is enhanced after it was added into solutions of the KW_5_ peptide, suggesting that KW_5_ can indeed exist in an aggregated β-sheet conformation. It is noteworthy that the maximum emission for the ThT fluorescence was observed when the KW_5_ peptide was dissolved in NaPB (sodium phosphate buffer, pH 7.2). Furthermore, the ThT fluorescence intensity increased at higher KW_5_ concentrations, and the self-aggregation of KW_5_ followed the order NaPB (pH 7.2) > PBS > 150 mM NaCl > 5 mM MgCI_2_ > NaPB (pH 5.2).

### 3.3. Fungicidal Activity of KW_n_ Peptides in the Presence of Sodium Azide

**Figure 3:**
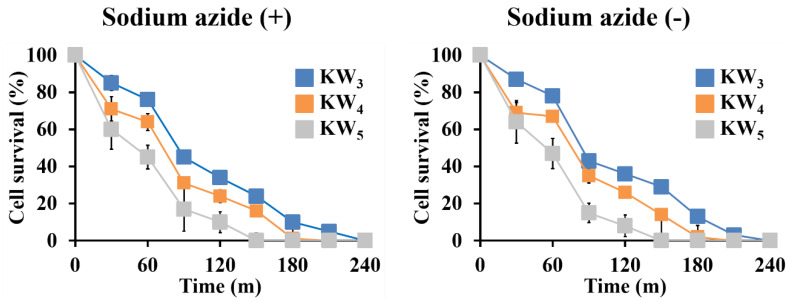
Time-kill-kinetics of the KW_n_ peptides against *C. albicans* in the absence and presence of sodium azide. The concentrations used were 32 μM for KW_3_, 8 μM for KW_4_ and 8 μM for KW_5_. Three biological replicates were run for each test sample, and the recorded values were averaged with the error bars representing the SD. For some data points no error bars can be seen due to the small SD.

We analyzed the time dependence of the fungicidal concentration (MFC) assay for these peptides, to compare their fungicidal actions. At their MIC values, the KW_n_ peptides completely eradicated *C. albicans* after 3 h (Figure 3). Our results also show that the longer KW_5_ peptide exhibited a faster eradication against *C. albicans* when compared to the shorter peptides. Addition of sodium azide can alter the lipid membrane potential in *C. albicans*, and it was also shown to inhibit the antimicrobial activity of Histatin 5 [39]. However, the fungicidal activity of the KW_n_ peptides against energy-depleted *C. albicans* was not inhibited in the presence of 5 mM sodium azide (Figure 3).

### 3.4. Cytotoxicity of the KW_n_ Peptides Against Mammalian Cells

**Figure 4:**
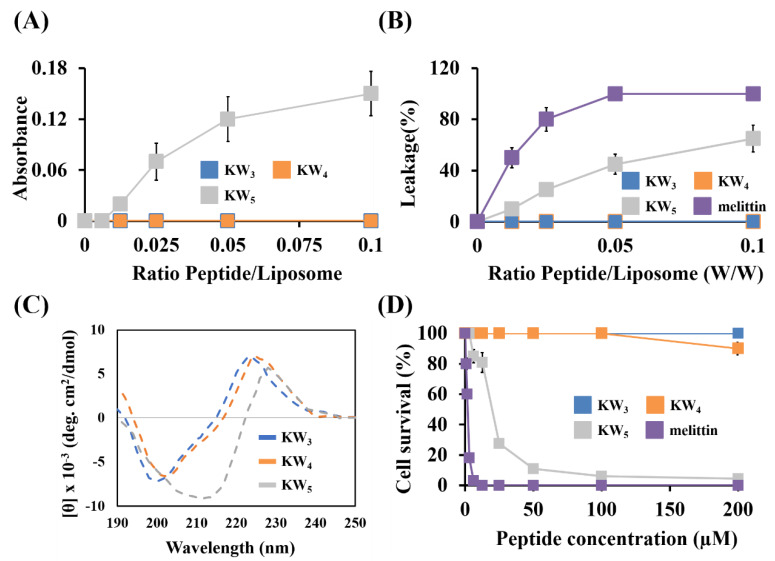
Cytotoxic activity of the KW_n_ peptides. (**A**) LUV aggregation as a function of peptide concentration. Absorbance was measured at 405 nm. (**B**) Calcein leakage in PC–CH–SM (1:1:1, *w*/*w*/*w*) vesicles. (**C**) CD spectra of peptides in the presence of PC–CH–SM (1:1:1, *w*/*w*/*w*) SUVs. (**D**) Cytotoxicity of the peptides measured with HaCaT cells using the MTT assay. Error bars represent the SD (the averaged value for each test sample from experiments performed in triplicate). No error bar appears for some data points due to the small SD.

We investigated the cytotoxicity of the KW_n_ peptides using liposomes mimicking normal eukaryotic membranes (PC–CH–SM (1:1:1, *w*/*w*/*w*)) as well as cultured HaCaT cells (Figure 4A–D). The membrane disruption activity or liposome fusion (aggregation) activity was tested for all of these peptides using LUVs with a 1:1:1 ratio of PC–CH–SM. The KW_3_ and KW_4_ peptides did not induce aggregation of this liposome (Figure 4A). Also, the KW_3_ and KW_4_ peptides did not cause calcein leakage from the eukaryotic membrane liposomes (Figure 4B). Indeed, these two experiments clearly indicated that KW_3_ and KW_4_ do not act as membrane disrupting peptides toward eukaryotic membrane mimetics. In contrast, the KW_5_ peptide induced significant aggregation of PC–CH–SM (1:1:1, *w*/*w*/*w*) LUVs (Figure 4A), while also inducing 60% calcein leakage from the same liposomes, indicating that KW_5_ can act as a lytic peptide. In this respect, KW_5_ somewhat resembles the well-known cytotoxic peptide melittin, which itself caused 90% calcein leakage under these conditions from the same liposomes (Figure 4B).

Next, the cytotoxicity of these peptides was determined against cultured HaCaT cells, an immortalized human keratinocyte cell line. The results show that melittin induced 100% cytotoxicity at a concentration of 12.5 μM. Among the KW_n_-series peptides, only the KW_5_ peptide showed cytotoxicity, but at a concentration above its MIC or MFC value, while the KW_3_ and KW_4_ peptides were not damaging to HaCaT cells even at concentrations as high as 100 μM (Figure 4D). Using CD spectroscopy, we also studied the secondary structure of these peptides in the presence of PC–CH–SM (1:1:1, *w*/*w*/*w*) liposomes. The KW_3_ and KW_4_ did not adopt any regular secondary structure, while KW_5_ adopted a distinct conformation, likely due to its aggregation in aqueous solution (Figure 4C). Overall, these data indicate that amongst this group of peptides, only KW_5_ has cytotoxic properties that can affect mammalian cells.

### 3.5. Inhibitory Effect of KW_4_ on the Formation of C. albicans Biofilms

**Figure 5:**
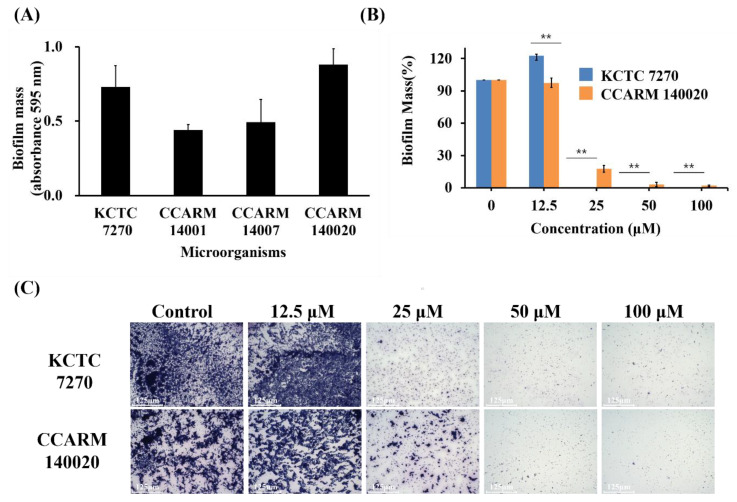
Inhibition of *C. albicans* biofilm formation by KW_4_. (**A**) Quantitative measurement of the biofilm formation for four strains. (**B**) Comparison of the inhibitory effect of KW_4_ addition on biofilm formation. (**C**) Microscopic analyses of biofilms formed by *C. albicans* stained with 0.1% crystal violet and the effects of treatment with KW_4_ at various concentrations. Columns indicate the means, bars show the SEM (*n* = 3). Scale bar = 125 μm. ** *p* < 0.01 versus control (two-tailed Student’s *t*-test).

The antibiofilm activity of the KW_4_ peptide was assessed against *C. albicans* strains. When comparing biofilm formation between the four strains listed in Table 1, two strains—*C. albicans* (KCTC 7270) and the fluconazole-resistant strain *C. albicans* (CCARM 140020)—formed a large biofilm mass in the wells (Figure 5A). Therefore, we selected these two strains for determining the antibiofilm activity of KW_4_. The results show that KW_4_ at a concentration of 25 μM caused more than 80% inhibition of biofilm formation (Figure 5B). Furthermore, microscopy revealed that crystal violet staining of the biofilm mass was significantly decreased by adding KW_4_ at a concentration of 25 μM when compared to untreated cells in both strains (Figure 5C). These findings indicate that KW_4_ has strong antibiofilm activity against biofilm-forming *C. albicans* strains.

### 3.6. Interaction of Peptides with Fungal Cell Wall Components and Fungal Membranes

**Figure 6:**
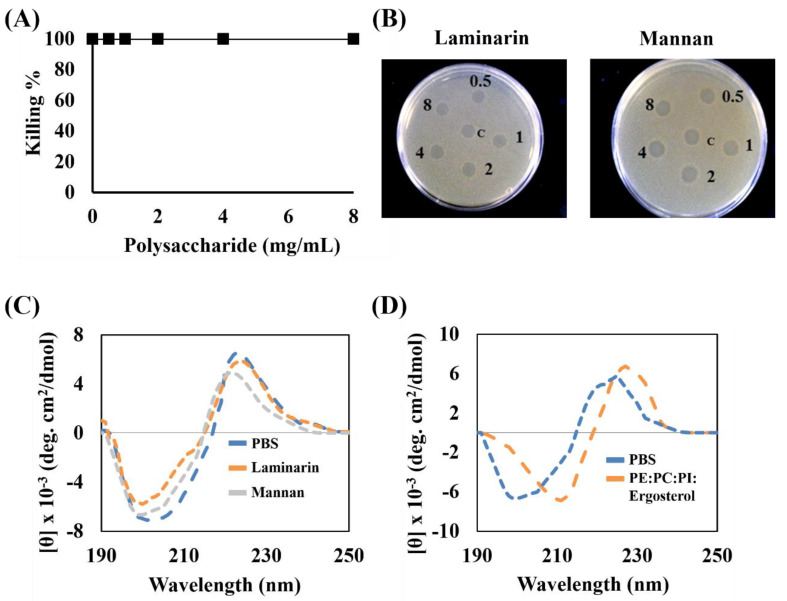
Binding of KW_4_ to cell wall components and liposomes mimicking fungal membranes. (**A**) The anticandidal activities of KW_4_ (8 μM) were measured at various concentrations of polysaccharides (data obtained for the two carbohydrates overlap in this graph). Three replicates for each test sample were performed, but error bars are not visible due to the small SD for each data point. (**B**) Radial diffusion assays in the presence of varying amounts of laminarin or mannan in the presence of KW_4_ in a final volume of 8 μL at 8 μM. The numbers represent the polysaccharide concentrations (mg/mL) of the mixture loaded in each well. (**C**) CD spectra recorded for 50 μM of KW_4_ in PBS and in the presence of mannan or laminarin. (**D**) CD spectra of KW_4_ recorded in the presence of PE–PC–PI–ergosterol (5:4:1:2, *w*/*w*/*w*/*w*) which mimic the outer leaflet of the membrane of *C. albicans*.

In order to determine whether KW_4_ can bind to typical fungal cell wall polysaccharides such as mannan and laminarin [47], we tested their potential influence using the microtiter plate and radial diffusion assays. It was found that the fungicidal activity of KW_4_ was not influenced by the addition of these polysaccharides at concentrations ranging from 0 to 8 mg/mL (Figure 6A,B). Next, the secondary structures of KW_4_ were analyzed in the presence of these polysaccharides by CD spectroscopy. These results revealed that KW_4_ displayed relatively small conformational changes that would be associated by binding to the polysaccharides laminarin or mannan (Figure 6C). Overall, the fungicidal experiments clearly indicated that these cell wall components do not play a role in the fungicidal activity of KW_4_ against *C. albicans*.

To further monitor the KW_4_ fungal lipid membrane interactions, we performed additional CD experiments in the presence of PE–PC–PI–ergosterol (5:4:1:2, *w*/*w*/*w*/*w*) liposomes mimicking fungal membranes (Figure 6D). A spectral change was clearly observed for the transition from aqueous solution to the membrane-bound state. While these data clearly confirm binding of the KW_4_ peptide to the fungal membrane mimetic, they provide little information about the secondary structure of the bound peptide, as the main peak observed between 225 and 230 nm originated from the Trp indole rings [48], and did not directly reflect the conformation of the peptide backbone.

### 3.7. Non-Permeabilizing Action of the KW_n_ Peptides in Fungal Membranes

**Figure 7:**
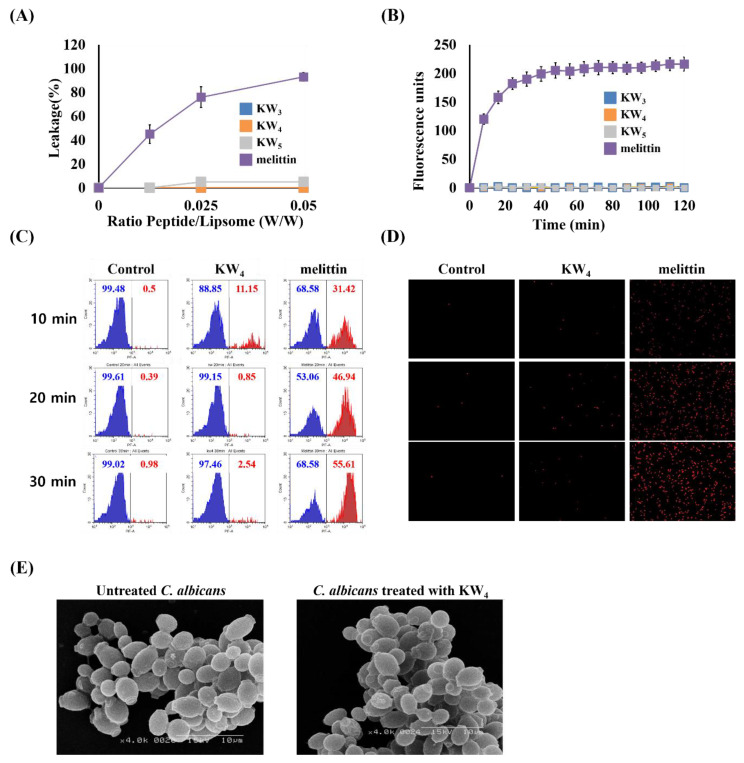
Lack of permeabilization of the cell membrane by the KW_n_ peptides. (**A**) Calcein leakage by peptide in PE–PC–PI–ergosterol (5:4:1:2, *w*/*w*/*w*/*w*) SUVs. (**B**) Analysis of membrane permeabilization caused by peptides using the SYTOX green dye. (**C**) Flow cytometry evaluation of the fungal membrane integrity using PI with KW_4_ (8 μM) or melittin (4 μM). (**D**) Fluorescence microscopy of *C. albicans* stained with PI after treatment with peptides at 1× MIC for 10, 20 and 30 min. (**E**) SEM image of *C. albicans* treated with KW_4_ at 1× MIC. The error bars in panels A and B represent the SD, which was calculated from the average obtained for experiments performed in triplicate.

To further investigate the mode of action of the KW_n_ peptide against *C. albicans*, we assessed its membrane permeabilization capabilities, by studying calcein leakage from PE–PC–PI–ergosterol (5:4:1:2, *w*/*w*/*w*/*w*) liposomes. At a ratio of 5:4:1:2 these liposomes mimic fungal membranes [49]. In this experiment, the control peptide melittin caused major (80%) calcein leakage from the PE–PC–PI–ergosterol liposomes, while KW_5_ caused only 5% calcein leakage. However, no calcein leakage was observed for the KW_3_ and KW_4_ peptides from these fungal liposomes even at the highest P/L ratio tested (Figure 7A). Furthermore, the integrity of the fungal membrane was examined by studying the distribution of the fluorescent dyes SYTOX green and propidium iodide (PI) after treatment with the peptides. Normally, these dyes do not penetrate into an intact cell, but if the fungal membrane is disrupted by AMPs or other antimicrobial agents then these dyes can enter the cells and form complexes with DNA that emit a higher level of fluorescence when compared to the dyes in aqueous solution. SYTOX green was added to the cells at a fixed concentration, then the cells were treated with peptide at different concentrations to monitor the time course of SYTOX green uptake into the cells for 120 min. It was found that the control peptide melittin caused complete membrane permeabilization within 35 min, whereas none of the KW_n_ peptides caused any dye influx (Figure 7B). Flow cytometry analysis was also performed to confirm the lack of membrane permeability of KW_4_ with fungal membranes. In contrast to the results obtained with KW_4_, melittin increased the PI uptake into the cells in a time-dependent manner (Figure 7C), clearly indicating that this positive control peptide causes membrane permeabilization. Finally, fluorescence microscopy was used to visualize propidium-iodide-stained DNA in the cells following peptide treatments in a concentration-dependent (Supplementary Figure S1) or a time-dependent manner (Figure 7D). Whereas melittin damaged the fungal cells and allowed the uptake of PI (as confirmed by the emergence of strong red fluorescence emissions), no fluorescence increase was detected when KW_4_ was used to treat *C. albicans* cells (Figure 7D). Subsequently, scanning electron microscopy (SEM) experiments also showed that KW_4_ at its MIC value did not damage *C. albicans*, as both the treated and untreated *C. albicans* cells had the same shape without any detectable changes in cellular morphology (Figure 7E). All these experiments strongly suggest that KW_4_ did not damage the fungal membrane (Figure 7A–E).

### 3.8. Membrane Binding Action of Peptides

As shown in Table 2, peptide binding and partitioning into lipid bilayers was also examined by measuring the Trp blue shift and the *K*_SV_ values, as measured in fluorescence spectroscopy and fluorescence quenching experiments, respectively [50]. A larger blue shift and lower *K*_SV_ values were observed in the presence of vesicles composed of PE–PC–PI–ergosterol (5:4:1:2, *w*/*w*/*w*/*w*) than for zwitterionic PC–CH–SM (1:1:1, *w*/*w*/*w*) vesicles (Table 2). These values directly indicate that the KW_n_ peptides partitioned into a more rigid region of the fungal liposome than the eukaryotic liposomes. In addition, all peptides (KW_3_, KW_4_ and KW_5_) had lower *K*_SV_ values, indicating that their Trp residues were partitioned into the hydrophobic core of the fungal liposomes (PE–PC–PI–ergosterol (5:4:1:2, *w*/*w*/*w*/*w*)) and suggesting that the indole sidechains of the Trp residues of these peptides were not accessible for the water-soluble quencher acrylamide. Moreover, better binding (larger blue shifts) and partitioning (lower *K*_SV_ values) were noted with increased peptide chain lengths in both liposomes. However, of the peptides tested, only KW_5_ bound and inserted into PC–CH–SM (1:1:1, *w*/*w*/*w*) liposomes, as a larger blue shift and a lower *K*_SV_ value (345 and 2.7 nm, respectively), were observed for this peptide when compared to KW_3_ and KW_4_. These results also agree with the outcome of the membrane disruption experiments, which showed that only KW_5_ perturbed the eukaryotic membrane mimetic PC–CH–SM (1:1:1, *w*/*w*/*w*).

### 3.9. Nucleic Acid-Binding Properties of KW_4_ and Observation of Peptide Action by Confocal Microscopy

**Figure 8:**
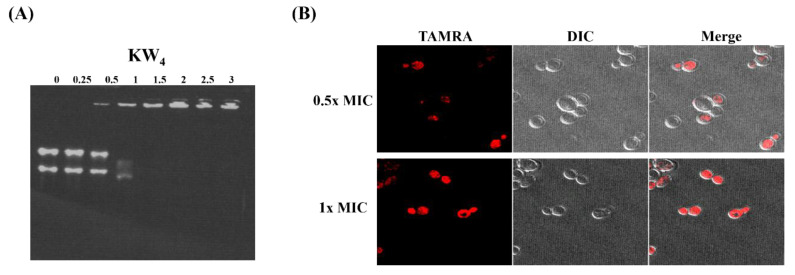
Morphological analysis of *C. albicans* and RNA binding of the KW_4_ peptide. The concentrations of KW_4_ range from 2.5 to 30 µg for this assay in order to create a different ratio with a fixed amount (10 µg) of RNA. (**A**) Gel retardation assay showing peptide binding to RNA obtained from *C. albicans,* the numbers above the figure indicate the ratio of the peptide and RNA concentrations. (**B**) Confocal laser-scanning microscopy (CLSM) images of *C. albicans* incubated with rhodamine-labeled KW_4_. The cells were incubated with 0.5× or 1× MIC for 10 min in PBS buffer. TAMRA (5-carboxytetra methyl rhodamine) and DIC (differential interference contrast microscopy) were merged in the right panel.

To evaluate the RNA-binding properties of the KW_4_ peptide, we performed a gel retardation assay [27]. Analysis of the band positions in the gel indicated that KW_4_ partially inhibited RNA migration at a ratio of 1, but fully blocked migration when KW_4_ was incubated with RNA at a ratio of 1.5 (Figure 8A). Experiments with the membrane-active AMP magainin II showed that it did not cause a band shift, while the AMP indolicidin, which can act intracellularly, did cause a similar band shift (see Supplementary Figure S2). These data clearly show that the KW_4_ peptide can interact with fungal RNA in vitro and possibly in vivo. To further support the idea that KW4 may enter the fungal cell and then bind to RNA, we visualized *C. albicans* that was incubated with rhodamine-labeled KW_4_ by confocal laser-scanning microscopy (CLSM). CLSM showed that the rhodamine labeled-KW_4_ peptide could penetrate into the cytoplasm of *C. albicans*, where RNA is present. Moreover, even at about half the MIC value of KW_4_ (4 µM), the peptide can already penetrate into the cytoplasm (Figure 8B). Overall, our results suggest that KW_4_ does not perturb the plasma membrane as part of its mechanism of action, but that nucleic acids in the cytoplasm or in the nucleus of fungal cells might be the target site for KW_4_ that gives rise to its fungicidal action against *C. albicans*.

## 4. Discussion

It has previously been reported that the KW_n_ and RW_n_ peptide series can have potent bactericidal activities, and that both these peptides can act on pathogenic bacteria through a membrane disruption mechanism [35,51]. Typically antimicrobial peptides need a proper balance of hydrophobicity and positive charge to specifically localize on the negatively charged bacterial membrane interface and disrupt the packing of lipids [35,51]. KW_4_ and KW_5_ both seem to have the proper balance, but the extra residues of KW_5_ do increase the propensity of this peptide towards disrupting mammalian membranes, reducing its selectivity. On the other hand, KW4 has a broad spectrum and selective activity against drug-resistant Gram-negative bacteria, drug-resistant Gram-positive bacteria and fungal *Fusarium* plant pathogens within a favorable concentration range of 2–32 µM [35,52]. In this work we have focused exclusively on the KW_n_ series because these have a lower activity towards mammalian host membranes when compared to the RW_n_ series. To date, it is not clear how the KW_n_ peptides can eradicate fungal cells. Therefore, in this contribution, we have focused much of our attention on a study of the mechanism of action of the KW_n_ peptides against the human pathogen *C. albicans*.

Here, we found that the antifungal activity of these peptides increases by extending the peptide chain length. We noted that the shortest KW_2_ tetrapeptide, consisting of only two Lys and two Trp amino acids, was inactive against fungal cells, while the KW_3_ hexapeptide already showed significant antifungal activity. Moreover, the two longer peptides, KW_4_ and KW_5_, showed approximately the same antifungal activity level against regular *C. albicans* strains. While KW_3_ had no antifungal activity against fluconazole-resistant *C. albicans* strains, the two longer peptides exerted a considerable antifungal activity against this strain (Table 1). These results clearly indicate that a threshold level of cationicity and hydrophobicity is required for the KW_n_ peptide series to act as antifungal peptides against *C. albicans*, including the multi-drug resistant *C. albicans* strains. Our results also show that peptide salt insensitivity improved with their increased chain lengths (Figure 2), which is consistent with a previous study [53]. Among all naturally occurring hydrophobic amino acids, Trp residues had the highest affinity for the lipid membrane interface, which might help to penetrate cationic peptides into the cytoplasmic membrane, acting together with the electrostatic attraction from AMPs towards the negatively charged bacterial cell surface [54]. Moreover, the anticandidal activity of the KW_n_ peptides was preserved at low pH (Figure 2D), suggesting that these peptides could potentially be used to inhibit *C. albicans* infections in different compartments of the human body (e.g., the skin, the vagina, chronic dental foci, etc.) that have acidic pH environments [55]. Furthermore, NaCl is an important environmental factor in the milieu surrounding human cells, where it can have varying concentrations. Our results demonstrate that the antifungal activity of longer KW_n_ peptides is preserved in the presence of relatively high NaCl concentrations. While the peptides could be considered to be a new class of antifungal agents, the longer KW_5_ peptide displayed some features that were different from the KW_3_ and KW_4_ peptides_._ Most notably, it displayed some cytotoxicity against human cells and was also found in an aggregated state in aqueous solutions. It seems that increasing the peptide length from KW_4_ to KW_5_ triggers self-association and peptide stacking, which in turn may have created its cytotoxic effects against the HaCaT keratinocyte cells (Figure 4). In a previous study we showed that KW_5_ can self-associate in PBS at increasing peptide concentrations [35]. Therefore, our results indicate that KW_4_ provides an optimum peptide length that can serve as a relatively noncytotoxic antifungal peptide. Moreover, KW_4_ showed potent antifungal activity even in rich media (data not shown) and in high salt conditions when compared to KW_3_. In addition, the synthetic KW_4_ octapeptide can be produced economically on a large scale.

In this work, we also determined the antibiofilm activity of the KW_4_ peptide against *C. albicans*. This was evaluated because *C. albicans* cells have been shown to form biofilms that protect them from antifungal therapeutics and from the host immune system [5], thereby making biofilm-forming *C. albicans* difficult to inhibit or eradicate. Our result show that the ability of *C. albicans* to form biofilms was almost completely inhibited by the KW_4_ peptide (Figure 5).

All these fungicidal actions persisted in the presence of the metabolic inhibitor sodium azide (Figure 3), suggesting that the fungicidal activity of these peptides is not dependent on an energy-dependent mechanism, unlike what has been reported for beta-defensins [56]. Also, our fungicidal data indicated that a longer time was needed for the peptides to exert their fungicidal activity when compared to that of a typical membranolytic peptide, which normally acts in a few minutes to complete its bactericidal activity. The longer time scale would be consistent with a different mechanism of action involving peptide entry into the *C. albicans* cells [35].

Because of its favorable properties, we selected the KW_4_ peptide for the majority of our studies of the mechanism of action. Compared to KW_3_ and KW_5_, it is an effective antifungal and antibiofilm agent, it has low salt sensitivity and is non-hemolytic [35,52]. Cell wall compounds can be essential for the action of antifungal peptides or proteins against pathogens [38,57,58]. A previous study has suggested that cell wall components strongly influence the internalization of histatin 5 in fungi [38]. Moreover, in another study it was emphasized that the NAD1 protein requires cell wall components for the permeabilization of the plasma membrane and its subsequent entry into target cells, thereby causing the eradication of fungal cells [58]. We examined the role of different cell wall polysaccharides in the activity of KW_4_ peptide; however, the presence of cell wall polysaccharides did not inhibit its fungicidal activity (Figure 6A,B), even though CD experiments showed some binding to these polysaccharides (Figure 6C). Be that as it may, our results clearly indicate that cell wall compounds do not play an essential role in the fungicidal activity of KW_4_ against *C. albicans*.

Normally the fungicidal action of AMPs would occur either (i) through disruption of the fungal membrane, (ii) by targeting intracellular components in fungal cells or (iii) through a combination of these events. Therefore, we examined the rate of membrane permeabilization using three different methods: (1) recording leakage of calcein from liposomes mimicking fungal membranes, (2) observing the entry of the SYTOX green dye, (3) noting the uptake of the propidium iodide dye into cells using flow cytometry and fluorescence microscopy. The calcein leakage data established that the KW_n_ peptide series were not fungal-membrane lytic agents even at very high peptide lipid ratios (Figure 7A). This observation is consistent with the outcome of the SYTOX green uptake assay, which indicated the absence of permeabilization of the fungal membranes (Figure 7B). These results are contrasted with those obtained using the membranolytic control peptide melittin, which caused calcein leakage from fungal liposomes [59]. Addition of the KW_4_ peptide also did not induce an influx of PI into *C. albicans* cells (Figure 7C,D), indicating again a non-membrane permeabilizing action for KW_4_. Moreover, a similar conclusion could be drawn from the SEM data, which showed that KW_4_-treated *C. albicans* cells maintain their overall shape and integrity (Figure 7E). These data suggest that the fungicidal action of KW_4_ likely involves other targets, possibly interacting with intracellular negatively charged polymers such as DNA and RNA.

It is conceivable that KW_4_ resembles the cellular uptake of cell-penetrating peptides that can act by direct translocation through cell membranes. In this process, the cell-penetrating peptides undergo a conformational change that is caused by peptide–membrane interactions [60,61,62]. We therefore studied the binding of the KW_n_ peptide series to fungal membrane mimetics by fluorescence and CD spectroscopy. Our results demonstrated relatively large blue shifts and small *K*_SV_ values for the KW_n_ series peptides, which suggests that these peptides partition into a more hydrophobic environment (Table 2). Also, the CD spectroscopy data provided evidence for the binding of KW_4_ to fungal membrane mimetics. Although binding to fungal membrane mimetics was demonstrated, no leakage was observed. In contrast, the KW_4_ peptide caused membrane leakage in prokaryotes [35]. This indicates that the KW_4_ peptide has an entirely different mechanism of action when acting on bacteria and fungi. In part, this is related to the contribution of cholesterol and ergosterol, which rigidify the membranes of eukaryotic and fungal cells. However, it is presently unclear how other lipid components may contribute to these two different actions of the KW4 peptide, and this would need to be investigated in future studies.

Once the peptide reaches the interior of a cell, possibly through disproportionation of toroidal pore structures [63], it can perform other functions such as binding to nucleic acids that in turn can give rise to the fungicidal activity of the peptide. To test this notion, we performed a gel retardation experiment which confirmed that a complex can be formed between KW_4_ and fungal RNA (Figure 8A). We speculate that KW_4_ can strongly bind to the phosphodiester bonds of RNA (and DNA) via its positively charged Lys side chains, and that the Trp indole ring can stack between the nucleotide bases in the RNA duplex. Similar cationic AMP DNA complexes have already been described in the literature [64,65]. For example, the cationic AMP HPA3NT3-A2 is known to interact with DNA and RNA, and this process is believed to contribute to its killing mechanism against microbes [64]. In another study, Lys- and Trp-rich puroindoline-derived peptides were shown to bind to DNA, thereby implicating an intracellular mechanism of action for these AMPs [65]. As in these other studies, the characterization of the KW_4_-RNA interactions (Figure 8A), makes it likely that KW_4_-DNA interactions can occur as well. Furthermore, the location of the fluorescently labeled KW_4_ peptide in live cells was examined by confocal microscopy, showing that the rhodamine-labeled KW_4_ peptide was internalized into the cytoplasm of *C. albicans* (Figure 8B). Such an internalization mechanism for KW_4_ into fungi is reasonable because the presence of negatively charged phospholipid headgroups in the fungal membrane would provide selectivity for the uptake of KW_4_. In fact, Lys side chains can interact with lipid phosphodiester groups through electrostatic and hydrogen bond interactions [66,67]. Trp residues are involved in the stabilization of the lipid-peptide complex, and the Trp indole ring has a unique ability to interact with the membrane interface [68,69]. Furthermore, previous studies have indicated that membrane interactions are important for short combinatorial Trp-rich peptides, and confocal microscopy results obtained for these peptides show that they can rapidly become localized in the cytoplasm of microbial cells, which suggests that they act on intracellular targets [34,70]. Therefore, our results support the idea that KW_4_ acts by targeting intracellular components such as RNA or DNA, which in turn are expected to inhibit intracellular nucleic acid and protein synthesis, ultimately leading to its fungicidal action.

In conclusion, our results confirm that increasing the length in the KW_n_ peptide series enhances their antifungal and cytotoxic activities. However, when compared to the KW_5_ peptide, KW_4_ had no detectable cytotoxic activity against the keratinocyte HaCat cell line at a concentration of 100 µM, making it potentially suitable for topical or wound healing applications. Furthermore, it maintained its antifungal activity in physiological salt concentrations and at low pH values. In addition, the KW_4_ peptide could effectively inhibit the *C. albicans* biofilm formation. We have demonstrated by CD and fluorescence spectroscopy that KW_4_ can bind to fungal membranes. However, KW_4_ did not induce calcein leakage from fungal liposomes, and various fluorescence (SYTOX green and PI) and microscopy experiments demonstrated that KW_4_ had a non-membranolytic activity against intact *C. albicans* cells. These observations correlated with the finding that the rhodamine-labeled KW_4_ peptide was translocated into the cytosol of *C. albicans*, where it could potentially eradicate fungal cells by interacting with intracellular targets such as RNA. Overall, our study indicates that the KW_4_ peptide acts on bacteria and fungi via membranolytic and non-membranolytic mechanisms, respectively. At the same time, KW_4_ had no hemolytic activity [35], and displayed no cytotoxic activity towards cultured human keratinocyte cells. The KW_4_ peptide therefore provides a suitable template for the development of new classes of antifungal or antibacterial drugs. In particular, its nonlytic mechanism of action makes it an attractive candidate as a future anticandidal agent.

## Figures and Tables

**Table 1:** MICs of the KW_n_ peptides against different *Candida albicans* strains.

Minimum Inhibitory Concentrations (µM)						
Strain	KW_2_	KW_3_	KW_4_	KW_5_	Melittin	Fluconazole
*C. albicans* (KCTC 7270)	>128	32	8	8	4	16
**Resistant strains**						
*C. albicans* (CCARM 14001)	>128	>128	32	8	16	>128
*C. albicans* (CCARM 14007)	>128	>128	32	16	8	>128
*C. albicans* (CCARM 140020)	>128	>128	32	8	8	>128
**Cytotoxicty**						
Cell survival ^a^ (%)	100	100	91	4	0	-
Hemolysis ^b^ (%)	0	0	8	71	100	-

^a^ Percent HaCaT (the human keratinocytes cell line) cell survival with 200 µM peptide. ^b^ Percent hemolysis of red blood cells with 200 µM peptide [35].

**Table 2:** Tryptophan emission maxima and ^a^ *K*_SV_ measured in PBS (pH 7.2) or in the presence of 200 μM PE–PC–PI–ergosterol (5:4:1:2, *w*/*w*/*w*/*w*) SUVs or 200 μM PC–CH–SM (1:1:1, *w*/*w*/*w*) SUVs.

Peptide λ_max_ Buffer (nm)		Blue Shift (nm)		*K*_SV_ (M^−1^) ^a^		
		PE–PC–PI–Ergosterol	PC–CH–SM	Buffer	PE–PC–PI–Ergosterol	PC–CH–SM
KW_3_	353	6	1	15	2.8	5.7
KW_4_	353	8	1	14	2.2	5.2
KW_5_	351	10	6	11	2.0	2.7

^a^*K*_SV_ is the Stern–Volmer constant.

## Supplementary Materials

Supplementary materials can be found at https://www.mdpi.com/2076-2607/8/5/758/s1.
