# Supplementary material for: Antifungal and Antibiofilm Activities and the Mechanism of Action of Repeating Lysine-Tryptophan Peptides against Candida albicans
Source: Microorganisms. 2020 May 18;8(5):758. doi: 10.3390/microorganisms8050758 (PMC7285485; doi:10.3390/microorganisms8050758)
Supplement: Supplementary file 1 [file microorganisms-08-00758-s001.pdf]

microorganisms

Supplementary Data

Antifungal and Antibiofilm Activities and the Mechanism of Action of Repeating Lysine-Tryptophan Peptides against *Candida albicans*

Gopal Ramamourthy [1,2,†], Jonggwan Park [3,†], Changho Seo [3], Hans J. Vogel [1] and Yoonkyung Park [2,4,*]

1 Biochemistry Research Group, Department of Biological Sciences, University of Calgary, Calgary, AB T2N 1N4, Canada; rgopal@ucalgary.ca (G.R.); vogel@ucalgary.ca (H.J.V.)
2 Department of Biomedical Science and BK21-Plus Research Team for Bioactive Control Technology, Chosun University, Gwangju 61452, Korea
3 Department of Bioinformatics, Kongju National University, Kongju 38065, Korea; for_quality@naver.com (J.P.); chseo@kongju.ac.kr (C.S.)
4 Research Center for Proteineous Materials, Chosun University, Gwangju 61452, Korea
* Correspondence: y_k_park@chosun.ac.kr Tel.: +82-62-230-6854; Fax: +82-62-225-6758.
† These authors contributed equally to this work.

Received: 23 March 2020; Accepted: 16 May 2020; Published: date

Table S1. MICs of the KW_n peptides against different non-*albicans* candida strains.

	Minimum inhibitory concentration (μM)					
**Strain**	**KW_2**	**KW_3**	**KW_4**	**KW_5**	**melittin**	**fluconazole**
C. catenulate	>128	16	8	8	8	16
C. rugosa	>128	32	8	8	8	16
C. melibiosica	>128	32	8	8	8	16
C. glabrata	>128	32	8	4	4	8
C. *intermedia*	>128	32	8	8	8	16

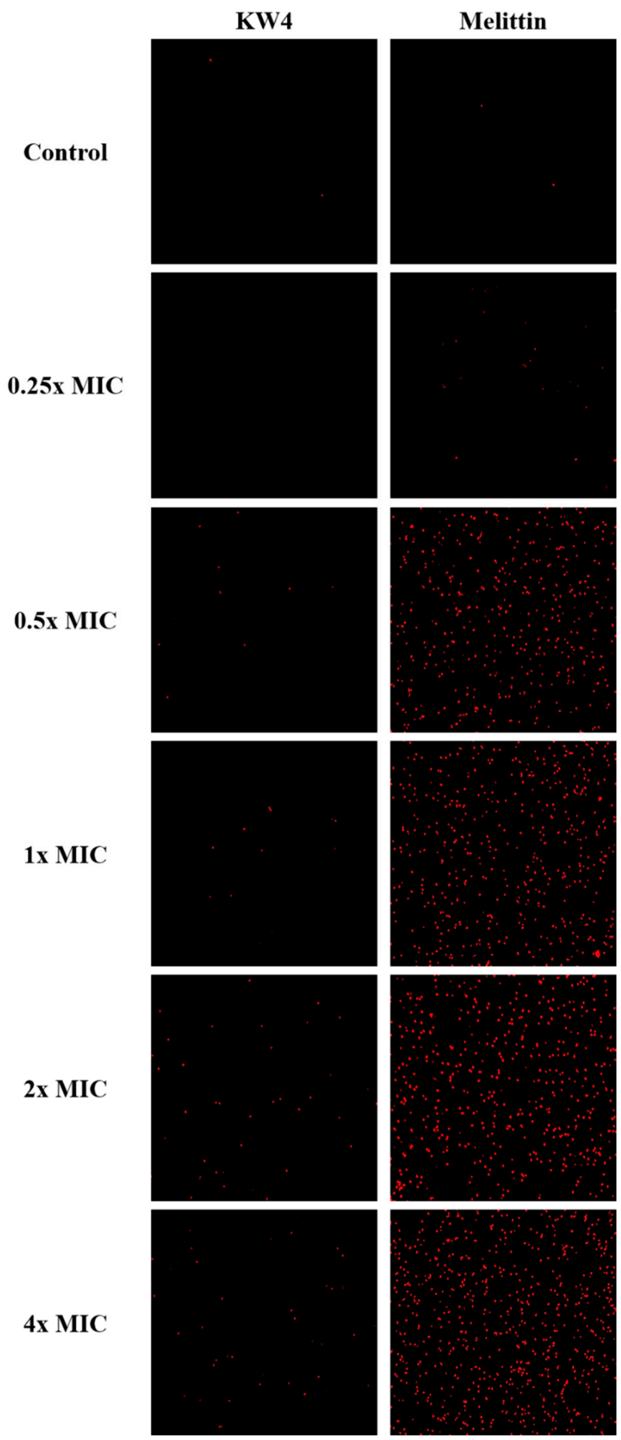

Figure S1. Fluorescence microscopy of *C. albicans* (2 x 10^6 cells/mL) in PBS buffer stained with Propidium Iodide (PI) after treatment with peptides at 0.25x MIC, 0.5x MIC, 1x MIC, 2x MIC and 4x MIC for 30 min. The visualization of *C. albicans* was achieved by using an EVOS FL Auto 2 imaging system (Invitrogen). Cells without added peptide served as a control. The control cytotoxic peptide melittin clearly causes influx of PI into the cells, in a concentration dependent manner, while KW_4 does not.

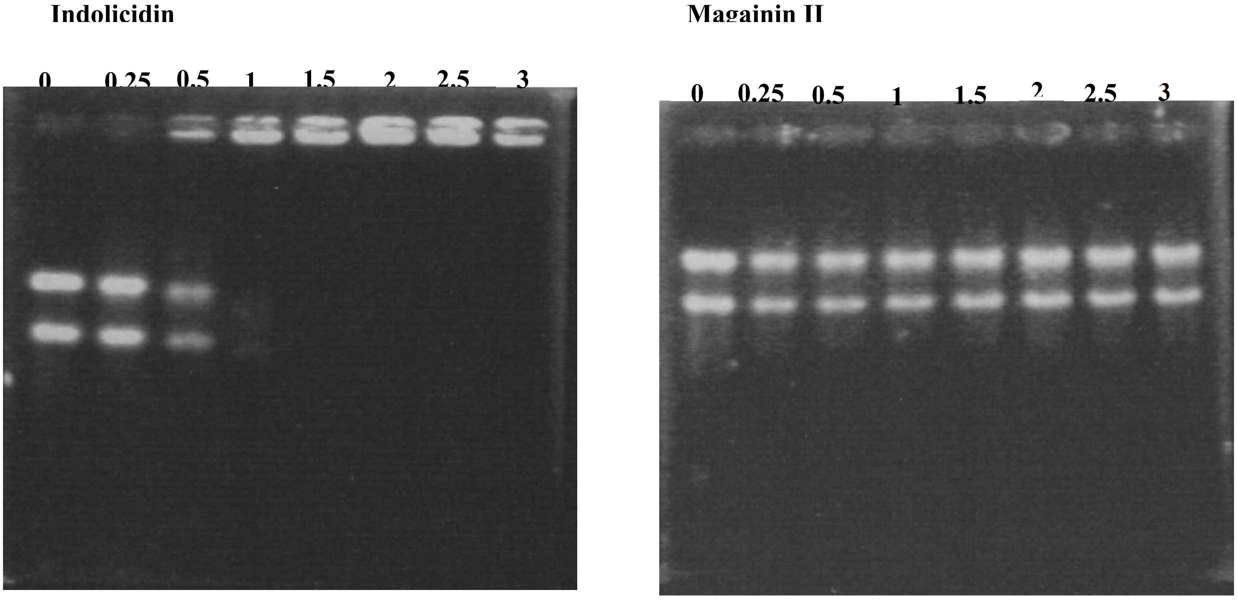

Figure S2. Gel retardation analysis of fungal RNA (10 µg) in the presence of the peptide. The mixture of peptide and RNA was incubated for 10 min at room temperature. Peptide was used at different concentrations such as 2.5, 5, 10, 15, 20, 25 and 30 µg in this study. The values mentioned at the top of the gels represent the peptide to RNA ratios. Indolicidin clearly causes a bandshift, while magainin II does not, even at higher concentrations.
